# Supplementary material for: Genome-Resolved Metagenomic Analyses Reveal the Presence of a Putative Bacterial Endosymbiont in an Avian Nasal Mite (Rhinonyssidae; Mesostigmata)
Source: Microorganisms. 2021 Aug 14;9(8):1734. doi: 10.3390/microorganisms9081734 (PMC8398770; doi:10.3390/microorganisms9081734)
Supplement: Supplementary file 1 [file microorganisms-09-01734-s001.zip › SupMaterial/Figure S1. Metabolic pathways found in the MAG.pdf]

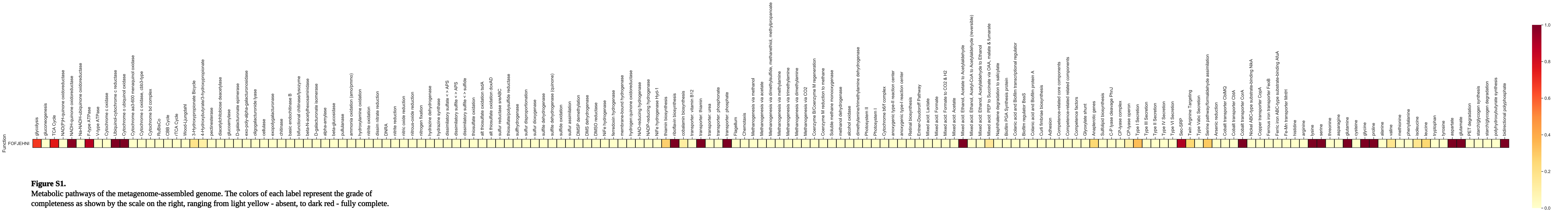

Figure S1.
Metabolic pathways of the metagenome-assembled genome. The colors of each label represent the grade of completeness as shown by the scale on the right, ranging from light yellow - absent, to dark red - fully complete.
